# Pseudo-depth-based deep neural network model for object detection

**DOI:** 10.1038/s41598-026-45310-w

**Published:** 2026-03-26

**Authors:** Si-Qi Li, Wei Feng, Bin Liu, Xin Tong, Qiang Li

**Affiliations:** 1 State Key Laboratory of Porous Metal Materials, School of Physical Science and Technology, Northwestern Polytechnical University, Xi’an, 710072 China; 2 School of Information Mechanics and Sensing Engineering, Xidian University, Xi’an, 710071 China; 3 Xi’an Key Laboratory of Advanced Remote Sensing, Xi’an, 710071 China; 4 Shaanxi Innovation Center for Multi-source Fusion Detection and Recognition, Xi’an, China; 5 Shanghai Aerospace Control Technology Institute, Shanghai, 201109 China

**Keywords:** Feature enhancement, Multispectral object detection, Pseudo-depth feature, Monocular depth estimation, Engineering, Mathematics and computing, Optics and photonics

## Abstract

Current machine learning methods only utilize the three-channel color features of optical images for computer visual tasks. However, the optical images only explicitly present information of RGB color and two-dimensional planar shape, where the third-dimensional spatial features are not fully exploited. This limitation restricts the potential improvement in recognition performance. To address this issue, we propose a detection scheme to enhance model’s detection capabilities based on four independent features by combining the pseudo-depth and the RGB features without adding any additional hardware sensors. The monocular depth estimation model is first used as a virtual depth sensor to extract the pseudo-depth features from input optical images. Then the fused Depth-RGB features are fed into the neural network model for object detection training and inference to enhance capability for extracting spatial features. Experiments show that the proposed method has improved the detection metric mAP$_{50}$ by 3.8 and 8.0 percentage points on the public M$^3$FD and COCO datasets, respectively. Notably, the scheme can be easily embedded into any machine learning models to definitely improve the detection performance.

## Introduction

Object detection serves as a core task in the field of computer vision, with extensive applications including autonomous driving, security surveillance, and scene analysis. Traditionally, these methods depend solely on RGB images to identify and locate targets, ignoring three-dimensional (3D) depth information. As a result, they only perform feature extraction and detection based on visible-light images. Over time, research has evolved into two mainstream directions: convolutional neural network (CNN)-based models and vision Transformer (ViT)-based models.

CNN-based detection frameworks are known for their efficiency in feature extraction while maintaining low computational requirements^1–4^. For example, YOLO^5^ predicts multiple bounding boxes and class labels in a single forward pass of the network. Unlike anchor-based methods, CenterNet ^6^ utilizes keypoint detection, resulting in a simpler and more streamlined process. Vision Transformer (ViT)-based networks, on the other hand, use an encoder-decoder architecture to capture global context. It allows for better modeling of long-range dependencies, though at the expense of increased computational demands^7–13^. Among these, the DETR^14^ eliminates many traditional pre- and post-processing steps by directly matching predictions to ground-truth labels using bipartite matching. Building on this, RT-DETR ^15^ designs a hybrid encoder to accelerate the inference speed via intra-scale and cross-scale feature interactions. It also adopts an uncertainty-minimized query selection mechanism to generate high-quality initial queries, which further improve the detection accuracy.

The detection methods discussed so far rely solely on two-dimensional (2D) RGB images for prediction. While these approaches have demonstrated promising results, they have notable limitations. RGB images provide detailed color and texture information of the target, but they lack explicit 3D spatial details^16^. As the key information of the third dimension, depth information offers crucial spatial and temporal cues that can significantly enhance object detection and recognition^17–19^. Without this data, the perceptual capabilities of deep networks remain restricted. To overcome this challenge, recent research has incorporated depth data to supplement spatial and structural information, leading to improved detection accuracy.

Researchers have explored multi-modal fusion between RGB and depth. Specifically, DMRANet^20^ utilizes residual connections to process 3D camera data, combining and fusing features from both RGB and depth streams at multiple levels. This approach enhances the complementary use of these two modalities. DETR3D^21^ adapts the Transformer-based detection framework from 2D to 3D, enabling the detection of objects from multiple views in three-dimensional space. The PETR framework^22^ performs direct 3D detection using multi-view images. It first divides the camera’s field of view into shared grid coordinates, then encodes these grid coordinates into 3D space and integrates them with image feature information, thereby improving the model’s spatial understanding ability. RadarPillars^23^ introduces a pillar-based detection network, that efficiently extracts features from radar point clouds by decomposing radial velocity data, enabling high-speed object detection. Beyond depth information, many studies have demonstrated that incorporating additional features can further enhance detection performance. For example, in remote sensing applications, combining infrared and hyperspectral data has proven effective in boosting detection accuracy^24–28^. Meanwhile, multi-scale fusion for multimodal data also contributes to improving the performance of object detection^29–31^.

Currently, 3D data are mainly obtained through methods such as structured light, LiDAR, and stereo vision systems^32–37^. However, these data often require additional processing, such as registration, before they can be effectively used in detection tasks. Although these multi-sensor methods effectively utilize 3D spatial features, they often face practical challenges such as high hardware costs, complex calibration procedures, limited sensor resolution, and increased technical complexity. These issues hinder their widespread deployment, especially in real-world scenarios where environmental conditions and budget constraints make multi-sensor systems impractical. To address these limitations, this work explores the possibility of incorporating depth information using only monocular RGB images. We propose a dedicated data preprocessing pipeline that generates pseudo-depth maps through monocular depth estimation model without needing additional distance sensor. This approach leverages the ability of monocular depth estimation to predict dense, reliable depth maps directly from RGB images, providing a practical and efficient foundation for enhancing object detection without relying on multiple sensors^38–40^.

We design a dual-branch network architecture to evaluate the effectiveness of the proposed method. Our approach begins by generating high-quality pseudo-depth maps from RGB images using a monocular depth estimation network^41–46^. The pseudo-depth maps, alongside the original RGB images, are fed into the dual-branch architecture consisting of two identical backbone. The feature maps produced by both branches are then concatenated and fused, allowing the model to learn combined information of the optical appearance and the pseudo-depth.

The proposed approach removes the requirement for additional depth sensors such as 3D cameras or LiDAR, thereby significantly lowering deployment costs and reducing technical complexity. By relying solely on monocular RGB images, our method utilizes both visual and pseudo-depth cues, offering a simple and cost-effective preprocessing pipeline that is easy to extend. Experiments on the M$^3$FD^47^ and COCO^48^ datasets show that incorporating pseudo-depth features improves detection accuracy compared to traditional RGB-based methods. Additionally, the model maintains a relatively simple architecture, making it straightforward to integrate into various network frameworks.

The main contributions of this paper are as follows:

1. We introduce a novel data preprocessing approach to enhance objection features that leverages pseudo-depth estimation from original RGB input images. The method has improved scene understanding capability and detection accuracy without additional sensors, making it low-cost and easy to implement;
2. We design a dual-branch feature fusion network that employs multi-scale concatenation and fusion strategies, which effectively reduces feature redundancies and facilitates the integration of pseudo-depth information and optical features;
3. Through extensive experiments on the M$^3$FD and COCO datasets, we demonstrate that incorporating pseudo-depth features can significantly improve object detection performance across multiple complex scenarios.

The rest of this work is organized as follows. Section “Method” describes the proposed pseudo-depth-based data preprocessing method and the design of the feature fusion network. Section “Experiments” details the experimental results and provides an analysis of their implications. Finally, Sect. “Summary and outlook” summarizes the conclusions of the study.

## Method

This section introduces a data preprocessing approach that combines pseudo-depth information with monocular RGB images to enhance object detection accuracy. The section elaborates on the fusion strategy, overall framework, and key technical details of the proposed method. To evaluate its effectiveness, YOLO11n is used as the baseline model.

### Extraction of spatial pseudo-depth features

Spatial depth information provides critical cues for object detection, particularly in scenarios involving occlusion, varying illumination, and cluttered backgrounds.

**Fig. 1:**
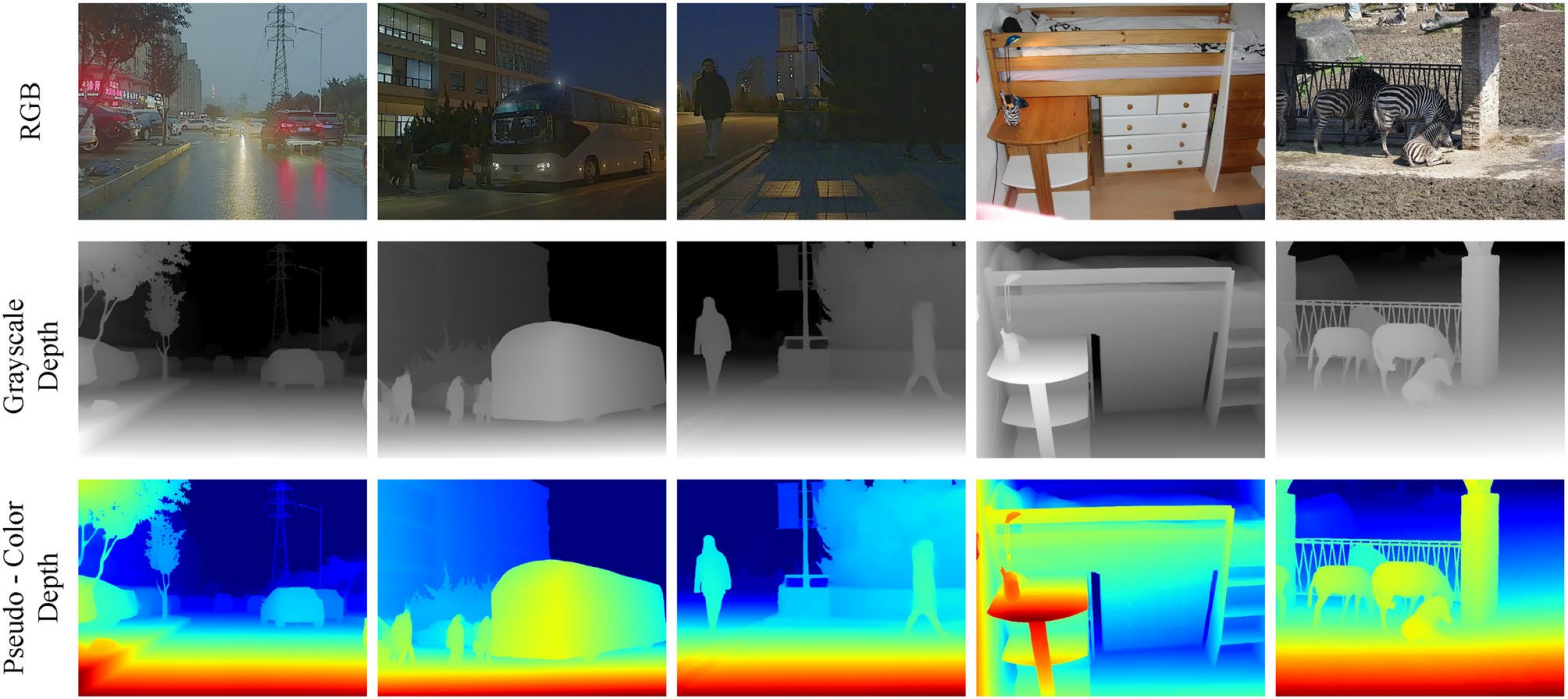
Example images from the M$^3$FD (left three columns) and COCO (right two columns) dataset. From top to bottom, the rows show the original RGB images, pseudo-depth maps generated by the monocular depth estimation (MDE) model, and their pseudo-color renderings, respectively.

As shown in Fig. 1, pseudo-depth maps can highlight object contours in low-light environments. Traditional methods for measuring depth include active sensors such as LiDAR and stereo camera systems. These distance sensors can deliver high-precision three-dimensional spatial data. However, the high costs, large physical sizes, and susceptibility to adverse environmental conditions have limited their widespread use in practical problems^49–52^. To address these limitations, we adopt a monocular depth estimation approach which infers the pseudo-depth information from only the corresponding RGB images. It allows us to model scene geometry without additional hardware.

**Fig. 2:**
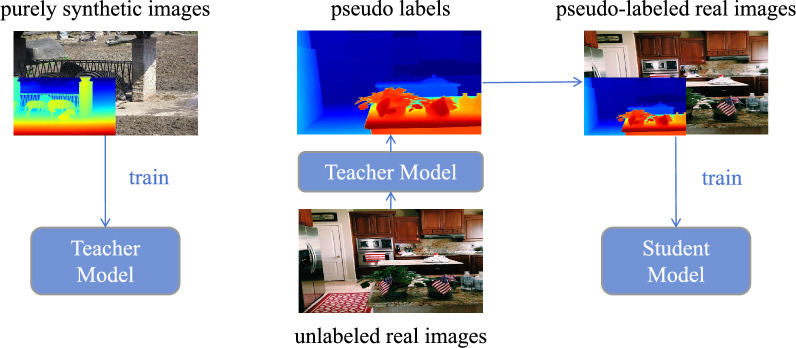
The overall architecture of Depth Anything V2^53^.

Here we use the Depth Anything V2^53^, an excellent monocular depth estimation model to validate our proposed method, whose network architecture is shown in Fig. 2. This model uses a two-stage training process. It first pre-trains on synthetic data to obtain the Teacher Model which then produces large-scale pseudo-annotated real images^54^. After being trained on these pseudo-annotated images, the model behaves quite well in complex environments. The model is also able to accurately detect details such as transparent objects and thin structures.

Its performance on standard benchmarks is shown in Table 1. The dense and continuous pseudo-depth maps produced by Depth Anything V2 contain pixel-level relative depth information. These maps complement the features of RGB images and improve scene understanding and object localization.

**Table 1:** Zero-shot relative depth estimation for Depth Anything V2^53^, where the $\delta _1$ metric representes proportion of pixel points with $\max (\frac{\hat{d}}{d}, \frac{d}{\hat{d}}) < 1.25$.

Model	KITTI	NYU-D	Sintel	ETH3D	DIODE
AbsRel	0.074	0.045	0.487	0.131	0.066
$\delta _1$	0.946	0.979	0.752	0.865	0.952

### Fusion of pseudo-depth and RGB features

To effectively leverage the complementary information from RGB images and pseudo-depth maps, this work employs a fusion strategy that utilizes parallel feature extraction networks^55,56^. Fusion methods are typically classified into early, middle, and late fusion based on the stage at which they combine features. Early fusion techniques, such as CrossFuse^57^ and DIVFusion^58^, merge raw data or low-level features directly, often leading to high computational costs and limited ability to capture modality-specific details. Conversely, late fusion^59^ approaches integrate high-level features or detection outputs, but they limit cross-modal interaction and collaboration.

**Fig. 3:**
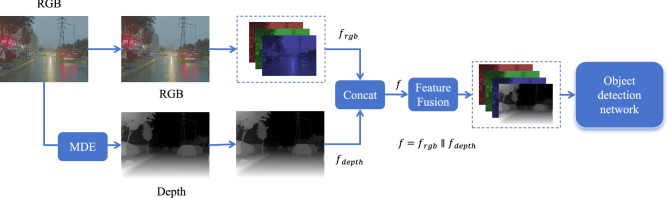
The overall structure of the experimental network is as follows. An RGB image is used to generate a pseudo-depth map via the MDE, which is then fed into the network through a dual-branch structure for feature fusion.

Middle fusion offers a balance by integrating semantic information at intermediate feature levels. This approach preserves modality-specific characteristics while enabling effective cross-modal interaction^60–63^. For example, Transformer-guided cross-modal fusion (CMF)^64^ can learn long-range dependencies, supporting both intra-modal and inter-modal feature integration. Similarly, MBNet^65^ employs modality-aware modules and feature alignment to enhance complementarity and reduce discrepancies during fusion. Considering these advantages, this work adopts a middle fusion strategy. The overall framework is shown in Fig. 3, where the feature maps of the RGB branch $f_{\text {rgb}}$ and the pseudo-depth one $f_{\text {depth}}$ are concatenated to produce the fused feature map *f*.

### Experimental architecture

The foundation of the system is built on the YOLO11n detection framework^66^ for feature fusion. In YOLO11, The backbone network extracts features at multiple scales, capturing both semantic and spatial information. It consists of convolutional and fusion modules, incorporating multi-scale pooling and attention mechanisms. This backbone produces three key feature maps, labeled $f^{(3)}$, $f^{(4)}$, and $f^{(5)}$, each with different strides. In the neck network, bidirectional feature fusion combines high-level and low-level spatial features. This facilitates effective multi-scale object detection. The network outputs three fused feature maps, $\hat{f}^{(3)}$, $\hat{f}^{(4)}$, and $\hat{f}^{(5)}$. Finally, the detection head converts these fused maps into detection results, providing the final predictions.

**Fig. 4:**
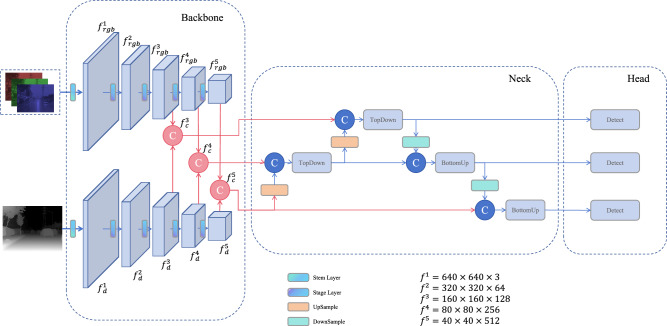
The network structure. The RGB and pseudo-depth features are concatenated at the $f^{(3)}$, $f^{(4)}$, and $f^{(5)}$ layer. Then the features are fused at multiple scales in the neck network. The three produced detection-scale features are finally used as input to the detection module.

The input to the proposed framework includes both RGB images and pseudo-depth maps generated from the original RGB ones through monocular depth estimation model. To enable effective feature fusion, the framework incorporates two core modules, namely, a dual-branch feature extraction module and a mid-level feature fusion module. The overall architecture is shown in Fig. 4.

The experimental framework features a dual-branch structure that processes RGB and pseudo-depth images separately. Each input is passed through an independent backbone network, designed similarly to the original YOLO11 backbone, to extract multi-layer features. The feature maps of layers $f^{(3)}$, $f^{(4)}$, and $f^{(5)}$ are concatenated to form fused feature maps, labeled as $f^{(3)}_{\text {c}}$, $f^{(4)}_{\text {c}}$, and $f^{(5)}_{\text {c}}$. The concatenation operation is formally defined as

1 $$\begin{aligned} f^{{(n)}}_{\text {c}} = f^{{(n)}}_{\text {rgb}} \parallel f^{{(n)}}_{\text {depth}}, \end{aligned}$$

where $n = 3, 4, 5$ and $f_{n}$ denotes the *n*-th feature layer.

These fused features undergo multi-scale processing in the detection head, which follows the same structure as the original YOLO11 head. For example, the output of the $f^{(3)}_{\text {c}}$ layer after passing through the detection head is denoted as $F^\text {head}_{f^{(3)}}$. The fusion formulation for $F^\text {head}_{f^{(3)}}$ is expressed as

2 $$\begin{aligned}&F_{f^{(4)}}^* = f^{(4)}_{\text {c}} \oplus g^{\text {Upsample}}(f^{(5)}_{\text {c}}, 2), \end{aligned}$$

3 $$\begin{aligned}&F_{f^{(3)}}^* = f^{(3)}_c \oplus g^{\text {Upsample}}\left( g^{\text {fusion}}(F_{f^{(4)}}^*, 512), 2\right) , \end{aligned}$$

4 $$\begin{aligned}&F_{f^{(3)}}^{\text {head}} = g^{\text {fusion}}(F_{f^{(3)}}^*, 256), \end{aligned}$$

where $g^{\text {Upsample}}(f, s)$ denotes upsampling *f* by a scale factor of *s*; $g^{\text {fusion}}(f, s)$ denotes compressing *f* to the target dimension *s* using the fusion module in YOLO11; $F^{\text {head}}$ represents the output of features at the corresponding scale in the head. The fused features at three scales are then fed into YOLO11’s detection module to produce the final detection results. Meanwhile, we performed similar modifications on YOLOv8 and RT-DETR by fusing their $f^{(3)}$, $f^{(4)}$, and $f^{(5)}$ feature layers. As illustrated in Fig. 4, the input feature dimensions of the five feature layers are annotated in the bottom-right corner of the figure. The red-highlighted sections (backbone and neck) represent operations that differ from the standard YOLO11n design in our setup. The remaining components, shown in blue, maintain the same structure as YOLO11n.

The loss function in YOLO11 comprises three components^66,67^: the object classification loss ($\text {Loss}_\text {cls}$), the localization loss ($\text {Loss}_\text {loc}$), and the distribution focal loss ($\text {Loss}_\text {dfl}$). The classification loss is used to control the correct class label for each object. It is computed by using the Binary Cross-Entropy (BCE)^68^. The localization loss evaluates the difference between the predicted bounding box and the actual bounding box. The localization loss consists of two parts, the center point coordinate loss and the width-height parameter loss. The former one evaluates the squared Euclidean distance between the predicted and ground-truth center coordinates of the bounding box,

5 $$\begin{aligned} \text {Loss}_\text {center} = \sum (\tilde{x}_i - x_i)^2 + (\tilde{y}_i - y_i)^2, \end{aligned}$$

while the latter one evaluates the difference between the predicted and the actual width-height parameters,

6 $$\begin{aligned} \text {Loss}_{w-h} = \sum \left( \sqrt{w_i} -\sqrt{\tilde{w}_i} \right) ^2 + \left( \sqrt{h_i} -\sqrt{\tilde{h}_i}\right) ^2, \end{aligned}$$

where the tilde variables denote the predicted ones. The distribution focal loss is introduced to address the issue of class imbalance in object detection and to improve the performance in dealing with small targets and difficult samples^69^. Formally, $\text {Loss}_\text {dfl}$ is defined as

7 $$\begin{aligned} \text {Loss}_\text {dfl}(P_i, P_{i+1}) = -\left[ (y_{i+1} - y) \log (P_i) + (y - y_i) \log (P_{i+1}) \right] , \end{aligned}$$

where

8 $$\begin{aligned} P_i = \frac{y_{i+1} - y}{y_{i+1} - y_i}, P_{i+1} = \frac{y_ - y_{i}}{y_{i+1} - y_i}, \end{aligned}$$

where *i* denotes the index of the discrete coordinate points; *y* denotes the ground-truth location value; $y_i$ and $y_{i+1}$ are two consecutive discrete points closest to *y*. The total loss is computed as a weighted sum of the three components:

9 $$\begin{aligned} \text {Loss}_{\text {total}} = {\lambda }_{\text {cls}} \text {Loss}_\text {cls} + {\lambda }_{\text {loc}} \text {Loss}_\text {loc} + {\lambda }_{\text {dfl}} \text {Loss}_\text {dfl}, \end{aligned}$$

where ${\lambda }_{\text {cls}}$, ${\lambda }_{\text {loc}}$, and ${\lambda }_{\text {dfl}}$ are balance weights that control the contributions of the classification loss, localization loss, and distribution focal loss, respectively. These three weight parameters are set to the standard values used in the YOLO11 framework.

The combined loss function provides multi-level supervision. It helps reduce class imbalance and improves detection accuracy, especially in complex environments.

## Experiments

We conducted experiments on COCO and M$^3$FD datasets to validate the effectiveness of our proposed method. We compared the performance of the RGB-only detection and our pseudo-depth fusion methods. The results show that the proposed scheme can significantly improve the detection performance on both datasets. The proposed method also remains stable across different training iterations.

### Experimental settings

In this work, we evaluate the effectiveness of the proposed algorithm using the datasets M$^3$FD and COCO. The M$^3$FD data is a multi-modal dataset comprising synchronized thermal infrared and visible light RGB images captured via binocular optical systems and infrared sensors. This dataset emphasizes multi-modal features across various complex scenarios and different pixel variations. The images are resolved at $1024\times 768$ pixels. Annotations cover six categories, ‘Person’, ‘Car’, ‘Bus’, ‘Motorcycle’, ‘Lamp’, ‘Truck’. The dataset is split such that $80\%$ of the images are randomly selected for training and the remaining $20\%$ for testing. COCO is a large dataset for object detection. It contains images from natural and complex daily scenes. COCO dataset includes 328,000 images and 2.5 million annotated instances. The annotations cover 80 object categories, such as ‘Car’, ‘Bottle’, and ‘Cat’. For the experiments, 20,000 images were randomly sampled as the training set and 2,000 ones were used as the test set.

To quantitatively evaluate the performance of the proposed method, here we employ the detection metrics $\textrm{F}_\textrm{1}$-score, mAP$_{50}$ (mean Average Precision at IoU=0.50) and mAP$_{50:95}$ (mean Average Precision over IoU thresholds from 0.50 to 0.95)^70^. The ${F}_{1}$-score is defined as

10 $$\begin{aligned} {F}_{1} = \frac{2 {P} {R}}{{P} + {R}}, \end{aligned}$$

which comprehensively considers both precision (*P*) and recall (*R*) with the same weight. In our experiments, the ${F}_{1}$-score is denotes the mean ${F}_{1}$-score across all categories. IoU measures the degree of overlap between a single predicted bounding box and a single ground-truth box. For the prediction box *A* and ground truth box *B*, the IoU is defined as

11 $$\begin{aligned} \textrm{IoU} = \frac{A \cap B}{A \cup B}. \end{aligned}$$

The metric Average Precision (AP) denotes the area under the precision-recall curve for each class. In practice AP is the precision averaged across all recall values between 0 and 1. For the *i*th category,

12 $$\begin{aligned} \textrm{AP}_{i} = \int _{0}^{1} P_{i}(R_{i}) \, \textrm{d}R_{i} = \sum _{k=0}^{n} P_{i}(k) \Delta R_{i}(k), \end{aligned}$$

and the Mean Average Precision (mAP) denotes the mean of AP across all *C* classes,

13 $$\begin{aligned} \textrm{mAP} = \frac{1}{C} \sum _{i=1}^{C} \textrm{AP}_i. \end{aligned}$$

The experiments are performed on a workstation equipped with an NVIDIA Tesla P40 GPU, utilizing the PyTorch 1.12 framework. All input images are resized to $640\times 640$ pixels using the letterbox method^71^. The model training employs the Stochastic Gradient Descent (SGD)^72^ optimizer with a batch size of 16. The first 3 epochs serve as warm-up with an initial learning rate of 0.005, followed by linear decay to a minimum of $5\times 10^{-5}$. Data augmentation techniques such as Mosaic^73^ and MixUp^74^ are employed to improve generalization and robustness.

### Comparison and discussion of the experiment results

This study uses YOLO11n as the baseline model to assess the effectiveness of the proposed approach. For comparison, the classic YOLOv8-n and RT-DETR models are also employed to evaluate the generalization capabilities of the proposed method. All training procedures are conducted from scratch with no pre-trained weights used. We compare the performance of the model when using only RGB images against the performance when fusing pseudo-depth information. The results, obtained on COCO and M$^3$FD datasets, are summarized in Table 2.

**Table 2:** Comparison of our proposed scheme on the YOLO11n, the YOLOv8-n and the RT-DETR detection models on the COCO and the M$^3$FD data.

Data	Model	Method	Paras(M)	Flops(G)	mAP$_{50}$(%)	mAP$_{50:95}$(%)	${F}_\text {1}$(%)
M$^3$FD	YOLO11n	RGB	2.6	6.3	73.7	47.8	73.1
		RGB-pD	3.8	9.3	**(+3.8)**77.5	**(+3.2)**51.0	75.7
	YOLOv8-n	RGB	3.0	8.1	77.4	50.7	86.5
		RGB-pD	4.4	11.3	**(+2.5)**79.9	**(+2.3)**53.0	78.7
	RT-DETR	RGB	41.9	125.7	82.5	53.9	79.3
		RGB-pD	66.3	194.0	**(+1.3)**83.8	**(+1.5)**55.4	81.8
COCO	YOLO11n	RGB	2.6	6.5	41.8	28.6	43.3
		RGB-pD	3.8	9.5	**(+8.0)**49.8	**(+7.1)**35.7	50.9
	YOLOv8-n	RGB	3.2	8.8	41.3	28.0	42.5
		RGB-pD	4.5	12.0	**(+8.0)**49.3	**(+7.2)**35.2	50.1
	RT-DETR	RGB	42.1	126.0	37.7	24.6	42.5
		RGB-pD	66.5	194.4	**(+8.5)**46.2	**(+7.8)**32.4	49.3

The results indicate that incorporating pseudo-depth features significantly enhances the model’s detection performance on both the M$^3$FD and COCO datasets. Specifically, in the YOLO11n based experiments, mAP$_{50}$ metric can improve 3.8 and 8.0 percentage points on the M$^3$FD and COCO dataset, respectively. As shown in the table, similar performance gains are also observed in experiments using YOLOv8 and RT-DETR frameworks.

Tables 3 and 4 show the category-specific detection results for the YOLO11n-based experiments on M$^3$FD and COCO datasets, respectively. The rows present AP$_{50}$ and AP$_{50:95}$ scores for each category on the M$^3$FD dataset; while six randomly chosen categories are shown for COCO dataset. Across all categories, the model incorporating pseudo-depth can consistently outperform the original one.

**Table 3:** Category-specific detection results on M$^3$FD.

Class		People	Car	Lamp	Bus	Motorcycle	Truck
AP$_{50}$(%)	RGB	64.1	88.1	85.9	71.8	56.7	75.7
	RGB-pD	67.5	89.9	87.4	74.0	63.7	82.5
AP$_{50:95}$(%)	RGB	33.9	63.6	68.1	36.1	33.2	52.2
	RGB-pD	36.2	65.4	71.9	39.0	35.7	58.1

**Table 4:** Category-specific detection results on COCO.

Class		Person	Airplane	Motorcycle	Fire Hydrant	Bear	Toilet
AP$_{50}$(%)	RGB	68.2	71.3	47.6	61.7	62.3	61.2
	RGB-pD	71.8	80.3	59.8	69.7	86.9	72.6
AP$_{50:95}$(%)	RGB	44.8	52.5	29.5	50.0	51.3	50.9
	RGB-pD	49.3	57.2	36.8	59.5	65.9	60.8

**Fig. 5:**
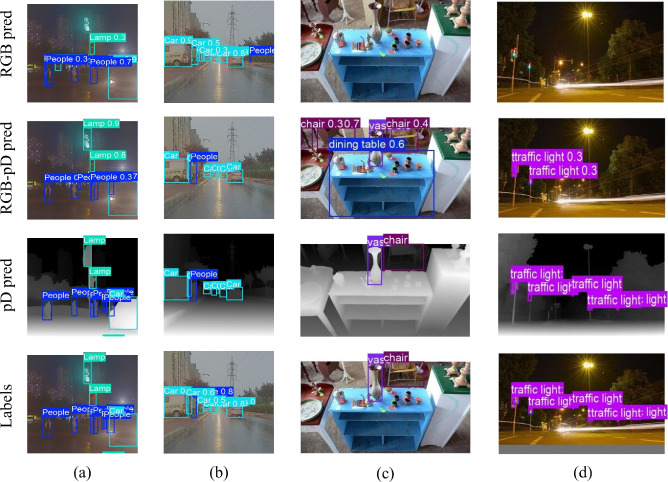
Visualization of the experimental results. The first two rows show the predicted results by the RGB-only features and the RGB fused with pseudo-depth features. The last two rows present the ground-truth annotations on the pseudo-depth images and the RGB images, respectively. The columns (**a**) and (**b**) show results on the M$^3$FD dataset, whereas  (**c**) and (**d**) show results on the COCO data.

In the first two rows of Fig. 5, we show the predicted results without and with fusing the pseudo-depth feature. Integrating pseudo-depth information helps the model define object boundaries more clearly. It also reduces background noise, which decreases missed detections. This is especially noticeable in low-light or cluttered scenes. For example, in the M$^3$FD sample (Fig. 5a), the RGB-only model missed a lamp in the upper middle part of the image because of low lighting at night. However, the pseudo-depth map clearly shows the lamp’s contours. The detector that uses pseudo-depth can accurately identify the target, even when it blends into the background. This improvement comes from the pseudo-depth map’s ability to enhance object edges. Depth-guided cues help distinguish targets from noisy backgrounds, thus improving localization and detection reliability. It is important to note that adding pseudo-depth processing increases the computational load by approximately 46%. Despite this, the significant performance gains show that this trade-off is acceptable for practical applications.

**Fig. 6:**
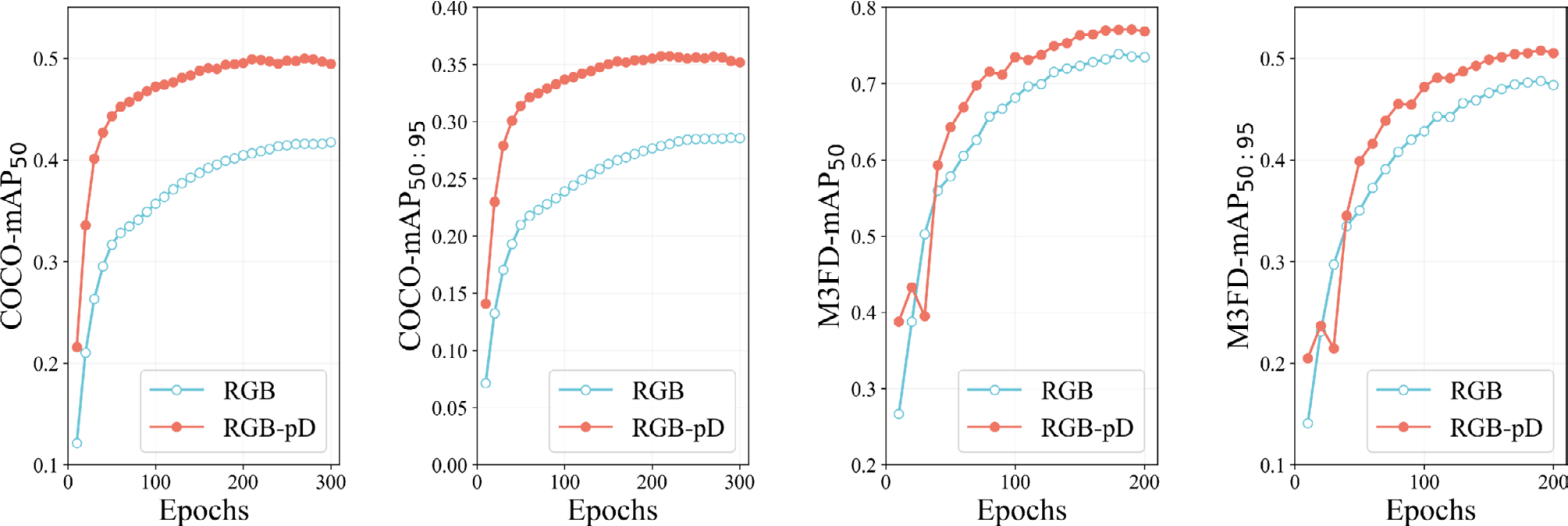
The metrics mAP$_{50}$ and mAP$_{50:90}$ versus the training epochs on the datasets M$^3$FD and COCO. The red solid (blue hollow) circle denotes the results based on the RGB-pD (RGB-only) features.

Figure 6 shows the changes in mAP$_{50}$ and mAP$_{50:95}$ during training on YOLO11n based model. Even at the early stages, incorporating pseudo-depth information significantly improves the detection performance. This benefit continues as the training progresses. The results demonstrate that the proposed method is effective and can produce performance gains even with limited training time. The performance difference between the two datasets is likely related to their scene characteristics. The COCO dataset contains many indoor and close-range images. In these scenes, depth estimation tends to be more accurate. This leads to a higher quality of pseudo-depth features and larger gains in detection performance. In contrast, the M$^3$FD dataset includes more outdoor scenes. Factors such as changing lighting conditions and long-distance targets make accurate depth estimation more difficult. As a result, the overall benefit of pseudo-depth fusion is limited.

**Table 5:** Inference efficiency of different models.

Model	Method	Latency(s, $\times 10^{-4}$)	FPS$_{bs = 1}$
YOLO11n	RGB	$102.5 \pm 16.0$	97.6
	RGB-pD	$152.4 \pm 27.1$	65.6
YOLOv8-n	RGB	$75.0 \pm 12.6$	133.4
	RGB-pD	$104.3 \pm 3.8$	95.8
RT-DETR	RGB	$262.1 \pm 15.5$	38.2
	RGB-pD	$351.3 \pm 43.4$	28.5

Table 5 shows the inference efficiency of several models. All experiments are conducted on an NVIDIA GeForce RTX 3090 GPU, with a batch size of 1, and the results are calculated based on 1000 test runs. The table indicates that the model with pseudo-depth integration experiences a reduction in inference efficiency compared to the original model. This decline is primarily due to the increased number of parameters resulting from the added branch, reflecting a trade-off between enhanced feature representation and computational speed.

### Visualization interpretation and ablation experiments

**Fig. 7:**
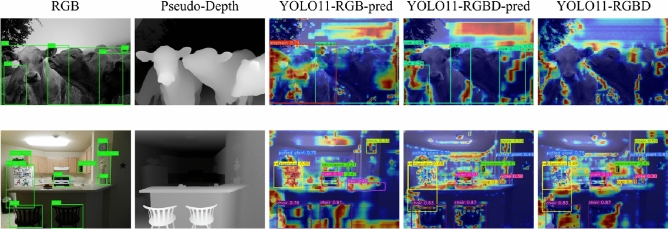
The GradCAM visualization of the two models on COCO dataset.

GradCAM is employed to visualize the prediction effects of the network. Figure 7 illustrates the GradCAM visualization results. Specifically, the first column shows the original images with manual bounding boxes, and the second column presents the corresponding pseudo-depth maps of the original images. The third and fourth columns display the visualization heatmaps of the detection heads for the original YOLO11 and the YOLO11 integrated with pseudo-depth information, respectively. The last column shows the visualization heatmaps of the $f^{(3)}_{\text {c}}$, $f^{(4)}_{\text {c}}$, and $f^{(5)}_{\text {c}}$ feature layers. It can be observed that after introducing the depth maps, the attention of the fusion layer is more concentrated on the boundaries of objects with obvious foreground-background relationships. Meanwhile, the attention of the detection head is accordingly focused on these regions, which improves the performance of object detection to a certain extent.

Since the comparison of RGB and RGB-pD already behaves like an ablation study, we design the ablation study by replacing the pseudo-depth maps by grayscale maps derived from the original RGB images to evaluate the effectiveness of the proposed module. Table 6 shows the ablation experimental results based on the YOLO11 framework.

**Table 6:** Ablation experiments of YOLO11 based framework on M$^3$FD and COCO datasets.

Dataset	Method	mAP$_{50}$(%)	mAP$_{50:95}$(%)
M$^3$FD	RGB	73.7	47.8
	RGB-pD	(+3.8)77.5	(+3.2)51.0
	RGB-gray	(+3.6)77.3	(+3.1)50.9
COCO	RGB	41.8	28.6
	RGB-pD	(+8)49.8	(+7.1)35.7
	RGB-gray	(+1.2)43.0	(+1.0)29.6

Results on COCO dataset confirm the validity of this ablation study. Compared with the RGB-gray model, the RGB-pD model achieves a 6.8 improvement in mAP$_{50}$, which indicates that introducing pseudo-depth information significantly boosts detection performance. On M$^3$FD dataset, the ablation study reveals only a modest performance difference between the proposed RGB-pD model and the RGB-gray model. This similarity in results may be due to the nature of the M$^3$FD dataset. The M$^3$FD dataset primarily features road scenes with relatively uniform but distant object characteristics. In these conditions, the obtained gray maps are much more similar with the pseudo-depth ones. The additional pseudo-depth information thus provides limited complementary benefit, resulting in a minor performance gap. The improvements on the COCO dataset demonstrate that the proposed module can substantially enhance detection performance in more diverse and complex data scenarios, providing strong evidence of its value for model optimization.

Meanwhile, we also observe that converting RGB images to grayscale before inputting them into the network leads to a noticeable improvement in detection performance. This improvement likely arises because grayscale input reduces the model’s reliance on color information, encouraging it to instead learn structural features such as edges and textures. Such features generally exhibit stronger generalization ability than color cues.

The experimental results confirm that the proposed pseudo-depth fusion method significantly enhances the performance of object detection models, particularly in scenarios where accurate depth estimation can be achieved. Moreover, the integration of pseudo-depth features offers several distinct advantages:

1. Enhance the perception of object boundaries and contours, reduce missed detections, and improves bounding box regression accuracy;
2. Boost the model robustness under challenging conditions such as low illumination and object occlusion;
3. Provide a low-cost, low-complexity scheme for integrating depth information, which is easy to implement and deploy.

## Summary and outlook

This work introduces a data preprocessing approach to improve RGB-based visual object detection based on estimated depth information. Unlike costly and complex real depth sensors, the method generates pseudo-depth maps from original RGB input images. We employ a dual-branch feature fusion strategy to concurrently extract and combine high-level features from both RGB and pseudo-depth inputs. Several comparative experimental results on the COCO and the M$^3$FD datasets confirm that the proposed scheme can consistently improve detection accuracy and robustness across multiple scenarios. The mAP metric improvement can reach 8 percentage points on COCO dataset. The advantages of the proposed scheme are twofold: (I) it provides a simple and low-cost solution without extra detection sensors; (II) it can be easily embedded into most machine learning models to definitely improve the detection performance.

On the hand, the results give new hint to further improve the visual performance besides the traditional updating in model architecture and data engineering. It reveals that though the deep neutral network can spontaneously extract complex abstract information from the original input, it can bring more benefit to feed the distilled features directly into the network for specific tasks. More independent features are supposed to improve the performance in machine learning tasks. Besides the passive RGB spectral features and the depth feature, the infrared information can behave as an independent active spectral feature to make positive effects. The (pseudo-)infrared features can be obtained similarly as the (pseudo-)depth one based on the deep neural network.

## Data Availability

The datasets generated and/or analysed during the current study are available in the GitHub repository with link https://github.com/htyb275/Pseudo-Depth-Detection.

## References

[1] Girshick, R., Donahue, J., Darrell, T. & Malik, J. Rich feature hierarchies for accurate object detection and semantic segmentation, in *2014 IEEE Conference on Computer Vision and Pattern Recognition*, pp. 580–587, (2014) 10.1109/CVPR.2014.81.

[2] Ren, S., He, K., Girshick, R. & Sun, J. Faster r-cnn: Towards real-time object detection with region proposal networks. *IEEE Trans. Pattern Anal. Mach. Intell.* **39**, 1137. 10.1109/TPAMI.2016.2577031 (2017). 27295650 10.1109/TPAMI.2016.2577031

[3] Dai, J., Li, Y., He, K. & Sun, J. R-fcn: object detection via region-based fully convolutional networks, in *Proceedings of the 30th International Conference on Neural Information Processing Systems*, NIPS’16, (Red Hook, NY, USA), p. 379–387, Curran Associates Inc., (2016).

[4] Law, H. & Deng, J. Cornernet: Detecting objects as paired keypoints, in *Computer Vision – ECCV 2018*, Ferrari, V., Hebert, M., Sminchisescu, C., & Weiss, Y., eds., (Cham), pp. 765–781, Springer International Publishing, (2018)

[5] Jiang, P., Ergu, D., Liu, F., Cai, Y. & Ma, B. A review of yolo algorithm developments. *Procedia Computer Sci.* **199**, 1066. 10.1016/j.procs.2022.01.135 (2022).

[6] Duan, K., Bai, S., Xie, L., Qi, H., Huang, Q., & Tian, Q. Centernet: Keypoint triplets for object detection, in *Proceedings of the IEEE/CVF International Conference on Computer Vision (ICCV)*, October, (2019).

[7] Zhu, X., Su, W., Lu, L., Li, B., Wang, X., & Dai, J. Deformable detr: Deformable transformers for end-to-end object detection, arxiv:2010.04159.

[8] Tong, X. et al. Meson Properties and Symmetry Emergence Based on the Deep Neural Network. *Chin. Phys. Lett.* **43**, 020201 (2026).10.1088/0256-307X/43/2/020201arxiv:2509.17093

[9] Caron, M., Touvron, H., Misra, I., Jégou, H., Mairal, J., Bojanowski, P., et al., Emerging properties in self-supervised vision transformers, in*Proceedings of the IEEE/CVF international conference on computervision*, pp. 9650–9660, 2021.

[10] Niu, Z., Zhong, G. & Yu, H. A review on the attention mechanism of deep learning. *Neurocomputing* **452**, 48. 10.1016/j.neucom.2021.03.091 (2021).

[11] Chen, Y., Chen, L., Xia, R., Yang, K. & Zou, K. Caat. Image super-resolution algorithm via channel attention and transformer. *Array* **28**, 100628. 10.1016/j.array.2025.100628 (2025).

[12] Meerits, S., Thomas, D., Nozick, V. & Saito, H. Fusionmls: Highly dynamic 3d reconstruction with consumergrade rgb-d cameras. *Comput. Visual Media.* **4**, 287 (2018).

[13] Chu, X., Deng, J., Ji, J., Zhang, Y., Li, H., & Zhang, Y. Oa-det3d: Embedding object awareness as a general plug-in for multi-camera 3d object detection: chu, X. et al., *International Journal of Computer Vision* 133 8022.(2025)

[14] Carion, N., Massa, F., Synnaeve, G., Usunier, N., Kirillov ,A., & Zagoruyko, S., End-to-end object detection with transformers, in *Computer Vision – ECCV 2020*, A. Vedaldi, H. Bischof, T. Brox and J.-M. Frahm, eds., (Cham), pp. 213–229, Springer International Publishing, (2020)

[15] Carion, N., Massa, F., Synnaeve, G., Usunier, N., Kirillov ,A., & Zagoruyko, S., End-to-end object detection with transformers, in *Computer Vision – ECCV 2020*, Vedaldi, A., Bischof, H., Brox, T., & Frahm, J.-M., eds., (Cham), pp. 213–229, Springer International Publishing, (2020)

[16] Cong, R. et al. Going from rgb to rgbd saliency: A depth-guided transformation model. *IEEE Trans. Cybern.* **50**, 3627. 10.1109/TCYB.2019.2932005 (2020). 31443060 10.1109/TCYB.2019.2932005

[17] Cong, R. et al. Cir-net: Cross-modality interaction and refinement for rgb-d salient object detection. *IEEE Trans. Image Process.* **31**, 6800. 10.1109/TIP.2022.3216198 (2022). 36288228 10.1109/TIP.2022.3216198

[18] Piao, Y., Rong, Z., Zhang, M., Ren, W. & Lu, H. A2dele: Adaptive and attentive depth distiller for efficient rgb-d salient object detection. *Proceedings of the IEEE Computer Society Conference on Computer Vision and Pattern Recognition* , 9057–9066 (2020).10.1109/CVPR42600.2020.00908.

[19] Wang, T., Zhu, X., Pang, J. & Lin, D. Fcos3d: Fully convolutional one-stage monocular 3d object detection. *Proceedings of the IEEE/CVF international conference on computer vision* , 913–922 (2021).

[20] Piao, Y., Ji, W., Li, J., Zhang, M., & Lu, H., Depth-induced multi-scale recurrent attention network for saliency detection, in *2019 IEEE/CVF International Conference on Computer Vision (ICCV)*, pp. 7253–7262, (2019), 10.1109/ICCV.2019.00735.

[21] Wang, Y., Guizilini, V.C., Zhang, T., Wang, Y., Zhao, H., & Solomon, J., Detr3d: 3d object detection from multi-view images via 3d-to-2d queries, in *Proceedings of the 5th Conference on Robot Learning*, Faust, A., Hsu D., & Neumann, G., eds., vol. 164 of *Proceedings of Machine Learning Research*, pp. 180–191, PMLR, 08–11 Nov, (2022), https://proceedings.mlr.press/v164/wang22b.html.

[22] Liu, Y., Wang, T., Zhang, X., & Sun, J., Petr: Position embedding transformation for multi-view 3d object detection, in *Computer Vision – ECCV 2022*, Avidan, S., Brostow, G., Cissé, M., Farinella, G.M., & Hassner, T., eds., (Cham), pp. 531–548, Springer Nature Switzerland, (2022).

[23] Musiat, A., Reichardt, L., Schulze, M., & Wasenmüller, O., Radarpillars: Efficient object detection from 4d radar point clouds, in *2024 IEEE 27th International Conference on Intelligent Transportation Systems (ITSC)*, pp. 1656–1663, IEEE, (2024).

[24] Song, H. et al. Cmkd-net: a cross-modal knowledge distillation method for remote sensing image classification. *Adv. Space Res.* **75**, 8515. 10.1016/j.asr.2025.04.009 (2025).

[25] Zhou, W., Cai, Y., Dong, X., Qiang, F. & Qiu, W. Adrnet-s*: Asymmetric depth registration network via contrastive knowledge distillation for rgb-d mirror segmentation. *Information Fusion* **108**, 102392 (2024).

[26] Ji, W., Li, J., Yu, S., Zhang, M., Piao, Y., Yao, S., et al., Calibrated rgb-d salient object detection, in *Proceedings of the IEEE/CVF Conference on Computer Vision and Pattern Recognition (CVPR)*, pp. 9471–9481, June, (2021).

[27] Song, H. et al. Symmetrical learning and transferring: Efficient knowledge distillation for remote sensing image classification. *Symmetry* **17**, 1002 (2025).

[28] Qi, C.R., Litany, O., He, K., & Guibas, L.J. Deep hough voting for 3d object detection in point clouds, in *Proceedings of the IEEE/CVF International Conference on Computer Vision (ICCV)*, October, (2019).

[29] Chen, Y., Xia, R., Yang, K. & Zou, K. Dual degradation image inpainting method via adaptive feature fusion and u-net network. *Appl. Soft Computing* **174**, 113010. 10.1016/j.asoc.2025.113010 (2025).

[30] Zhang, J., Yang, J., Qin, Y., Xiao, Z. & Wang, J. Mgnet: Rgbt tracking via cross-modality cross-region mutual guidance. *Neural Netw.* **190**, 107707. 10.1016/j.neunet.2025.107707 (2025). 40554301 10.1016/j.neunet.2025.107707

[31] Zhang, J., Zhang, S., Li, D., Wang, J. & Wang, J. Crack segmentation network via difference convolution-based encoder and hybrid cnn-mamba multi-scale attention. *Pattern Recog.* **167**, 111723. 10.1016/j.patcog.2025.111723 (2025).

[32] Shi, S., Wang, X., & Li, H. Pointrcnn: 3d object proposal generation and detection from point cloud, in *Proceedings of the IEEE/CVF Conference on Computer Vision and Pattern Recognition (CVPR)*, June, (2019).

[33] Zhang, H., Jiang, H., Yao, Q., Sun, Y., Zhang, R., Zhao H., et al. Detect anything 3d in the wild, in *Proceedings of the IEEE/CVF International Conference on Computer Vision (ICCV)*, pp. 5048–5059, October, (2025).

[34] Yang, L. et al. Bevheight++: Toward robust visual centric 3d object detection. *IEEE Transactions on Pattern Analysis and Machine Intelligence* (2025). 10.1109/TPAMI.2025.3549711 40067721

[35] Zhang, H. et al. Test-time correction: An online 3d detection system via visual prompting. *IEEE Trans. Pattern Anal. Mach. Intell.* **48**, 3666. 10.1109/TPAMI.2025.3642076 (2026). 41364566 10.1109/TPAMI.2025.3642076

[36] Yang, L. et al. Sgv3d: Toward scenario generalization for vision-based roadside 3d object detection. *IEEE Transactions on Intelligent Transportation Systems* (2025).

[37] Simony, M., Milzy, S., Amendey, K., & Gross, H.-M. Complex-yolo: An euler-region-proposal for real-time 3d object detection on point clouds, in *Proceedings of the European conference on computer vision (ECCV) workshops*, pp. 0–0, (2018).

[38] Weng, X., & Kitani, K. Monocular 3d object detection with pseudo-lidar point cloud, in *Proceedings of the IEEE/CVF International Conference on Computer Vision (ICCV) Workshops*, Oct, (2019).

[39] Chen, Z. et al. Graph-detr4d: Spatio-temporal graph modeling for multi-view 3d object detection. *IEEE Trans. Image Process.* **33**, 4488 (2024). 39093681 10.1109/TIP.2024.3430473

[40] Xie, Q., Lai, Y.-K., Wu, J., Wang, Z., Zhang, Y., Xu, K., et al. Mlcvnet: Multi-level context votenet for 3d object detection, in *Proceedings of the IEEE/CVF Conference on Computer Vision and Pattern Recognition (CVPR)*, June, 2020.

[41] Ranftl, R., Lasinger, K., Hafner, D., Schindler, K. & Koltun, V. Towards robust monocular depth estimation: Mixing datasets for zero-shot cross-dataset transfer. *IEEE Trans. Pattern Anal. Mach. Intell.* **44**, 1623. 10.1109/TPAMI.2020.3019967 (2022). 32853149 10.1109/TPAMI.2020.3019967

[42] Wang, J., Lin, C., Sun, L., Liu, R., Nie, L., Li, M., et al., From editor to dense geometry estimator, arxiv:2509.04338.

[43] Oquab, M., Darcet, T., Moutakanni, T., Vo, H.V., Szafraniec, M., Khalidov, V., et al., Dinov2: Learning robust visual features without supervision, *Transactions on Machine Learning Research* (2024) .

[44] Zhou, Y., & Tuzel, O. Voxelnet: End-to-end learning for point cloud based 3d object detection, in *Proceedings of the IEEE conference on computer vision and pattern recognition*, pp. 4490–4499, (2018).

[45] Liang, M., Yang, B., Wang, S., & Urtasun, R., Deep continuous fusion for multi-sensor 3d object detection, in *Proceedings of the European conference on computer vision (ECCV)*, pp. 641–656, (2018).

[46] Yang, Z., Sun, Y., Liu, S., Shen, X., & Jia, J. Std: Sparse-to-dense 3d object detector for point cloud, in *Proceedings of the IEEE/CVF international conference on computer vision*, pp. 1951–1960, (2019).

[47] Liu, J., Fan, X., Huang, Z., Wu, G., Liu, R., Zhong, W., et al. Target-aware dual adversarial learning and a multi-scenario multi-modality benchmark to fuse infrared and visible for object detection, in *Proceedings of the IEEE/CVF Conference on Computer Vision and Pattern Recognition*, pp. 5802–5811, (2022).

[48] Lin, T.-Y., Maire, M., Belongie, S., Hays, J., Perona, P., Ramanan, D., et al. Microsoft coco: Common objects in context, in *Computer Vision – ECCV 2014*, D. Fleet, T. Pajdla, B. Schiele and T. Tuytelaars, eds., (Cham), pp. 740–755, Springer International Publishing, (2014).

[49] Park, D., Ambrus, R., Guizilini, V.C., Li, J., & Gaidon, A. Is pseudo-lidar needed for monocular 3d object detection?, *2021 IEEE/CVF International Conference on Computer Vision (ICCV)* 3122.(2021)

[50] Ma, X., Liu, S., Xia, Z., Zhang, H., Zeng, X., & Ouyang, W. Rethinking pseudo-lidar representation, in *Computer Vision – ECCV 2020*, A. Vedaldi, H. Bischof, T. Brox and J.-M. Frahm, eds., (Cham), pp. 311–327, Springer International Publishing, (2020).

[51] Liu, Z., Tan, Y., He, Q. & Xiao, Y. Swinnet: Swin transformer drives edge-aware rgb-d and rgb-t salient object detection. *IEEE Trans. Circuits Syst. Video Technol.* **32**, 4486. 10.1109/TCSVT.2021.3127149 (2022).

[52] Zhang, H., Koh, J.Y., Baldridge, J., Lee, H., & Yang, Y., Cross-modal contrastive learning for text-to-image generation, in *Proceedings of the IEEE/CVF conference on computer vision and pattern recognition*, pp. 833–842, (2021).

[53] Yang, L., Kang, B., Huang, Z., Zhao, Z., Xu, X., Feng, J., et al. Depth anything v2, in *Advances in Neural Information Processing Systems*, A. Globerson, L. Mackey, D. Belgrave, A. Fan, U. Paquet, J. Tomczak et al., eds., vol. 37, pp. 21875–21911, Curran Associates, Inc., (2024), 10.52202/079017-0688.

[54] Tian, Y., Fan, L., Chen, K., Katabi, D., Krishnan, D., & Isola, P. Learning vision from models rivals learning vision from data, *2024 IEEE/CVF Conference on Computer Vision and Pattern Recognition (CVPR)* (2023) 15887.

[55] Bai, X., Hu, Z., Zhu, X., Huang, Q., Chen, Y., Fu, H., et al., Transfusion: Robust lidar-camera fusion for 3d object detection with transformers, in *Proceedings of the IEEE/CVF conference on computer vision and pattern recognition*, pp. 1090–1099, (2022).

[56] Li, Z. et al. Bevformer: Learning bird’s-eye-view representation from lidar-camera via spatiotemporal transformers. *IEEE Trans. Pattern Anal. Mach. Intell.* **47**, 2020. 10.1109/TPAMI.2024.3515454 (2025). 10.1109/TPAMI.2024.3515454 40030479

[57] Li, H. & Wu, X.-J. Crossfuse: A novel cross attention mechanism based infrared and visible image fusion approach. *Inf. Fusion* **103**, 102147. 10.1016/j.inffus.2023.102147 (2024).

[58] Tang, L., Xiang, X., Zhang, H., Gong, M. & Ma, J. Divfusion: Darkness-free infrared and visible image fusion. *Inf. Fusion* **91**, 477. 10.1016/j.inffus.2022.10.034 (2023).

[59] Chen, H., Li, Y. & Su, D. Multi-modal fusion network with multi-scale multi-path and cross-modal interactions for rgb-d salient object detection. *Pattern Recognition* **86**, 376. 10.1016/j.patcog.2018.08.007 (2019).

[60] Deevi, S.A., Lee, C., Gan, L., Nagesh, S., Pandey, G., & Chung, S.-J., Rgb-x object detection via scene-specific fusion modules, in *2024 IEEE/CVF Winter Conference on Applications of Computer Vision (WACV)*, pp. 7351–7360, (2024), 10.1109/WACV57701.2024.00720.

[61] Guo, X., Zhou, W. & Liu, T. Contrastive learning-based knowledge distillation for rgb-thermal urban scene semantic segmentation. *Knowledge-Based Syst.* **292**, 111588. 10.1016/j.knosys.2024.111588 (2024).

[62] Ma, J. et al. Multiscale sparse cross-attention network for remote sensing scene classification. *IEEE Trans. Geosci. Remote Sens.* **63**, 1. 10.1109/TGRS.2025.3525582 (2025).

[63] Wan, D., Lu, R., Fang, Y., Lang, X., Shu, S., Chen, J., et al., Yolov11-rgbt: Towards a comprehensive single-stage multispectral object detection framework, arxiv:2506.14696.

[64] Qingyun, F., Dapeng, H., & Zhaokui, W., Cross-modality fusion transformer for multispectral object detection, arxiv:2111.00273.

[65] Zhou, K., Chen, L., & Cao, X., Improving multispectral pedestrian detection by addressing modality imbalance problems, in *Computer Vision – ECCV 2020*, A. Vedaldi, H. Bischof, T. Brox and J.-M. Frahm, eds., (Cham), pp. 787–803, Springer International Publishing, (2020).

[66] Jocher, G., & Qiu, J., Ultralytics yolo11, (2024).

[67] Khanam, R., & Hussain, M., Yolov11: An overview of the key architectural enhancements, arxiv:2410.17725.

[68] Lin, T.-Y., Goyal, P., Girshick, R., He, K., & Dollar, P., Focal loss for dense object detection, in *2017 IEEE international conference on computer vision (ICCV)*, IEEE International Conference on Computer Vision, pp. 2999–3007, IEEE; IEEE Comp Soc, (2017), 10.1109/ICCV.2017.324.

[69] Li, X., Wang, W., Wu, L., Chen, S., Hu, X., Li, J., et al., Generalized focal loss: Learning qualified and distributed bounding boxes for dense object detection, in *Advances in Neural Information Processing Systems*, H. Larochelle, M. Ranzato, R. Hadsell, M. Balcan and H. Lin, eds., vol. 33, pp. 21002–21012, Curran Associates, Inc., 2020, https://proceedings.neurips.cc/paper_files/paper/2020/file/f0bda020d2470f2e74990a07a607ebd9-Paper.pdf

[70] Cartucho, J., Ventura, & Veloso, M., Robust object recognition through symbiotic deep learning in mobile robots, in *2018 IEEE/RSJ International Conference on Intelligent Robots and Systems (IROS)*, pp. 2336–2341, (2018).

[71] Redmon, J., & Farhadi, A., Yolov3: An incremental improvement, *arXiv preprint* arXiv:1804.02767 (2018) .

[72] Mustapha, A., Mohamed, L., & Ali, K., An overview of gradient descent algorithm optimization in machine learning: Application in the ophthalmology field, in *Smart Applications and Data Analysis*, Hamlich, M., Bellatreche, L., Mondal A., & Ordonez, C., eds., (Cham), pp. 349–359, Springer International Publishing, (2020).

[73] Bochkovskiy, A., Wang, C.-Y., & Liao, H.-Y.M., Yolov4: Optimal speed and accuracy of object detection, *arXiv preprint* arXiv:2004.10934 (2020) .

[74] Zhang, H., Cisse, M., Dauphin, Y.N., & Lopez-Paz, D., mixup: Beyond empirical risk minimization, arxiv:1710.09412.

